# Achieving across-laboratory replicability in psychophysical scaling

**DOI:** 10.3389/fpsyg.2015.00903

**Published:** 2015-07-02

**Authors:** Lawrence M. Ward, Michael Baumann, Graeme Moffat, Larry E. Roberts, Shuji Mori, Matthew Rutledge-Taylor, Robert L. West

**Affiliations:** ^1^Department of Psychology and Brain Research Centre, University of British Columbia, VancouverBC, Canada; ^2^Career Centre, University of the Fraser Valley, AbbottsfordBC, Canada; ^3^Interaxon, TorontoON, Canada; ^4^Department of Psychology, McMaster University, HamiltonON, Canada; ^5^Department of Informatics, Kyushu UniversityFukuoka, Japan; ^6^Cognitiva Information Science Institute, GatineauQU, Canada; ^7^Department of Cognitive Science, Carleton University, OttawaON, Canada

**Keywords:** psychophysical scaling, power function exponents, constrained scaling, loudness, psychological measurement

## Abstract

It is well known that, although psychophysical scaling produces good qualitative agreement between experiments, precise quantitative agreement between experimental results, such as that routinely achieved in physics or biology, is rarely or never attained. A particularly galling example of this is the fact that power function exponents for the same psychological continuum, measured in different laboratories but ostensibly using the same scaling method, magnitude estimation, can vary by a factor of three. Constrained scaling (CS), in which observers first learn a standardized meaning for a set of numerical responses relative to a standard sensory continuum and then make magnitude judgments of other sensations using the learned response scale, has produced excellent quantitative agreement between individual observers’ psychophysical functions. Theoretically it could do the same for across-laboratory comparisons, although this needs to be tested directly. We compared nine different experiments from four different laboratories as an example of the level of across experiment and across-laboratory agreement achievable using CS. In general, we found across experiment and across-laboratory agreement using CS to be significantly superior to that typically obtained with conventional magnitude estimation techniques, although some of its potential remains to be realized.

## Introduction

One of the hallmarks of the success of the “scientific method” in achieving understanding of, and possibly some small bit of control over, the natural world is the precise replicability of the results of scientific experiments. Typically an experimental result is not accepted as definitively demonstrated until one or more replications of it have occurred in different laboratories. Repeated failures to replicate usually discredit a result, regardless of its potential importance. Although in psychology in general such replications and their failures are not given the status they receive in other fields, such as physics, nonetheless they are important and are often done in the context of extensions of basic results or reinterpretations arising from conflicting results. Therefore it is important that there be both agreed upon criteria for replication, and also precise enough measurement of the relevant variables that those criteria can be applied. The former problem has been and is being addressed to some extent (e.g., Dixon and O’Reilly, 1999; Killeen, 2005; Open Science Framework, 2015), but the latter problem remains a difficult one. Surprisingly, this is true not only in fields such as social psychology, or personality, where measurement of psychological variables has a venerable but thorny tradition. It is true also even in psychophysics, arguably the most quantitatively advanced area of psychology.

In psychophysics, and in its applications in other areas, there are two major types of measurement. The first relies on experimental protocols that allow manipulations of physical variables to be “reflected” back from an experimental participant into the physical world, where the participant’s response to a stimulus is measured by counting (e.g., proportion of hits and false alarms – signal detection theory), or measured on a physical continuum such as time (e.g., response time), or by the level of some physical variable such as sound intensity, light intensity, etc. required to reach a certain performance criterion (Laming, 1986). The second type of measurement involves participants’ reporting directly on the magnitude of a sensory or other subjective experience such as similarity, confidence, and so forth, using a scale either of their own devising but with some constraints (e.g., Stevens, 1975), or a typical but informal scale such as a category scale from 1 to 10, 0 to 5, etc. Quantitative convergence across laboratories of results using the first kind of measurement is limited by sampling variability, experimental technique, and conceptual issues, but it is not limited significantly by measurement of the dependent variable(s) itself, as all measurement is either counting or done on a consensual physical scale (e.g., seconds, millivolts, candelas/m^2^) with very small measurement error. In spite of many successes (e.g., Marks, 1974a), however, the second kind of measurement, including especially psychophysical scaling on which this paper focuses, has not yet achieved nearly the degree of reliability and precision required to demonstrate quantitative convergence across different laboratories and experimenters.

Some indication of the magnitude of the problem is given by considering how the exponents of power functions relating the perceived loudness of a sound to physical sound intensity vary between individuals and across experiments. West et al. (2000) noted that variability in these exponents between individuals within an experiment was about as large as that of average exponents across experiments, with ratios of highest to lowest individual exponent in a single experiment of 3 or greater not uncommon, and even in some cases reaching as high as six. Variability of this magnitude suggests that the criteria adopted by individual subjects to characterize their perceptual responses may be idiosyncratic to an extent that compromises the meaning of individual comparisons, undermining the goal of psychophysical testing. This problem is amplified if the experimental aim is to characterize clinical populations such as those suffering from pain, tinnitus, or similar phenomena for the magnitude of their perceptual experience, or to contrast studies conducted at different sites and in different environments for the effectiveness of interventions aimed at treating these conditions. West et al. (2000) showed that this unwanted variation of scaling exponents across individuals with normally functioning sensory systems could be substantially reduced by training observers to use a standard scale on a standard sensory continuum and then, while keeping them calibrated on that scale, having them judge, on the same, calibrated scale, stimuli other than those used in training. This was accomplished by interleaving, in a strictly alternating sequence, trials on the trained, standard continuum with feedback (recalibration trials) with trials on the novel continuum without feedback (test trials). They named this technique *constrained scaling* (hereafter CS).

Ward and Baumann (2009) subsequently used CS to measure psychophysical functions for perceived loudness in a group of 14 individuals suffering from tinnitus and varying degrees of high frequency hearing impairment above 2 kHz. Exponents of power functions were determined for a 1 kHz trained tone, for a test tone of 500 Hz, and for a test tone corresponding to the subject’s tinnitus frequency. Among subjects with comparatively better hearing (*n* = 7) ratios of the largest to the smallest exponent were 1.46 for the 1 kHz trained tone, 1.71 for the test tone of 500 Hz, and 1.43 for the test tone corresponding to the tinnitus frequency. Among the poorer hearing subjects (*n* = 7) the corresponding ratios were similar (1.46, 2.14, and 1.53, respectively). These ratios reflected the magnitude of individual differences within the groups and appeared to be notably lower than those obtained with conventional magnitude estimation (CME) in normal hearing subjects (West et al., 2000). CS also found that the mean exponent obtained at the tinnitus frequency for the poorer hearing subjects (1.08) was twice that obtained for the better hearing subjects (0.50), uncovering hyperacusis in the former group.

The findings of West et al. (2000) and Ward and Baumann (2009) support the view that CS reduces between-subject variability in the magnitude estimation of perceived loudness and gives individual comparisons to which perceptual meaning can be assigned. Unaddressed, however, is whether CS can achieve the same degree of reduction of between-subject variability in different laboratory environments and reduce between-laboratory variation as well, both of which would be expected if perception is validly measured by CS. The present study was undertaken to answer these questions. To this end we compared the results obtained from nine different experiments conducted in four different laboratories to assess the level of between-experiment and cross-laboratory agreement achievable with CS when measuring perceived loudness. We also contrasted the results obtained with a training method of brief duration intended for clinical use with that of a more lengthy training method favored by laboratory experiments. In general, we found across experiment and across-laboratory agreement using CS to be significantly superior to that typically obtained with CME techniques, for both the brief and extended CS procedures. Although some of the potential of CS remains to be realized, application to other psychophysical continua and to practical problems of perceptual assessment in the clinic and industry appears to be warranted.

In the following we begin by presenting a more detailed history of the challenge of psychophysical measurement and the rational of CS. The results of the cross experiment and cross-laboratory investigations are then reported.

## Background and Rationale for Constrained Scaling

Several authors have described the difficulties involved in obtaining quantitative convergence in psychophysical scaling (e.g., Marks, 1974b; Ward, 1991; Laming, 1997). Imagine if the charge of the electron had a range of values that depended on who was doing the measurement experiment, or if the gas constant or the speed of light were not “constant” but depended on which investigator was writing about them. This problem actually did occur early in the history of physics, for example in the measurement of temperature (e.g., Middleton, 1966), but it was solved by the adoption of consensual, standard, scales for the measurement of the basic physical variables (e.g., Ellis, 1966). Unfortunately, this problem continues to plague psychophysical scaling, which is arguably one of the most successful techniques by which sensations and other conscious experiences are measured (e.g., Marks, 1974a). It finds its most dramatic manifestation in the unwanted variability of exponents of psychophysical power functions measured in different laboratories and even in different experiments in the same laboratory. Although Stevens (1975) argued that canonical exponent values should be adopted for all of the sensory continua, it has not been the case that the values he suggested could be achieved by every investigator, despite attempts to use the same methods and stimuli. A striking demonstration is the report by Marks (1974b) of average power function exponents ranging from 0.37 to 0.80 for the loudness of 1000 Hz pure tones, measured in various laboratories by direct ratio scaling techniques. Thus, if a theory, based on psychological, physiological and physical considerations, predicted that the exponent for loudness of a 1000 Hz tone should be 0.6 (e.g., Zwicker, 1982), that theory would be disconfirmed by the majority of scaling experiments reported to date, although the average exponent over all such experiments is indeed around 0.6 (Marks, 1974b).

Poulton (1989) attempted to classify and model all of the various kinds of bias that affect such judgments and presumably give rise to the unacceptable level of variability of power function exponents (and other properties). Others, e.g., Laming (1997), have suggested that such variation is the source of major interpretational problems with direct scaling results. Yet others, e.g., Lockhead (1992), have suggested that the attempt to achieve canonical psychophysical scales is fundamentally misguided because of profound contextual effects that influence all judgments of sensory or other experiential magnitude. Still, such scales do have vast usefulness, both in designing buildings, assessing environmental impact, and other applied contexts, and also in informing fundamental theories (e.g., Norwich, 1993). Therefore we have taken the approach that achieving canonical scales is a worthwhile goal. We should emphasize here that we are *not* talking about discovering the “true” exponent values for various psychological continua, but rather about adopting canonical exponent values that are consistent with basic psychophysical results, that make the expression of these results in the form of empirical laws simple and elegant, and that are consistently replicable across experiments and laboratories.

Constrained scaling is our solution to the problem of achieving canonical scales. As we mentioned above, CS involves training observers to use a *standard scale* on a *standard sensory continuum* and then, while keeping them calibrated on that scale, having them judge, on the same, calibrated scale, stimuli other than those used in training. To explain the technique further, consider that all non-standard stimuli must be judged *without* feedback, *because the sensation magnitudes arising from these stimuli are unknown*. The intent of CS is to require participants to use a standardized response scale to describe their sensation magnitudes, to induce a standardized meaning of the response scale values (i.e., *to use the same “meter stick”*), *not* to decide in advance what they will experience, or to constrain them to give the same exponents to the non-standard stimuli that they were trained to give to the standard stimuli. The training relates their experiences on a standard continuum to the standard response scale so that they use the numbers of the standard response scale in a consistent way relative to those experiences. Other, non-standard stimuli are judged in relation to the feedback-labeled experiences on the standard continuum. So, for example, a response of “10” to a non-standard stimulus means that the participant has experienced a sensation magnitude that is (approximately) the same as that induced by the standard stimulus they learned to call “10.” Across-participant consistency thus depends primarily on the degree of similarity of participants’ sensory experiences on the standard continuum. To the extent that these experiences are similar, and that their experiences on the test continua are as well, they will produce the same results on the test continua. If their experiences on the test continua differ, their results will differ. These differences will be interpretable as “true” experiential differences if participants have remained calibrated on the standard continuum. If their results on the standard continuum differ, as for example for those with sensory deficits, then all bets are off: the training will fail to produce convergence across participants and any differences on test continua will be meaningless. West et al. (2000) addressed all of these issues and provided data showing that CS is both more reliable than conventional techniques and also valid in that it does not distort basic psychophysical relationships such as the ratio of power function exponents across continua. We next briefly review their results in this regard.

West et al. (2000) trained observers to use a response scale for loudness of 1000 Hz pure tones closely related to Stevens’ sone scale, *S* = 10.6 *P* ^0.60^, in which sones, *S*, are a power function of sound pressure (*P*) with an exponent of 0.6. West et al. (2000) used *R* = 16.6 *P*^0.60^, where *R* is the required response, and responses could range from 0 to 100. West et al. (2000) used this slightly revised scale because on it the response to a 100 dB stimulus is about 100, giving a wider response range than is available for sones (40 dB is 1 sone and 100 dB is 63.9 sones). The conversion of responses on the West et al. (2000) scale to sones is *S* = 0.638 *R*. They then had observers judge 65 Hz tones *without feedback* on this same scale as described earlier, reproducing the usual finding that power function exponents are substantially larger for low frequencies than they are for 1000 Hz, and doing so for *every* individual observer and with extremely little between-observer variability in the relation between exponents. Interestingly, the exact quantitative relationship between exponents at various sound frequencies is still uncertain, partly because of the problem this paper addresses, that of inter-laboratory variability. West et al. (2000)’s result of a ratio of about 0.7 between the exponent for 1000 Hz and that for 65 Hz accords closely with the value of 0.64 reported by Ward (1990) and that of Schneider et al. (1972) for similar frequencies (1100 and 100 Hz, ratio = 0.69), although it is somewhat different from that implied by an equation suggested by Marks (1974a) based on results from several other laboratories (about 0.52). West et al. (2000) also found a similar ratio between 1000 and 65 Hz exponents when they trained participants to a standard scale in which the 1000 Hz exponent was 0.30 (ratio = 0.69), but not when they trained participants to a standard scale with an exponent of 0.90 (ratio = 0.92). In the latter case participants also did not closely reproduce the training exponent for 1000 Hz tones (0.75 versus training exponent of 0.90), and although they produced a higher exponent for the 65 Hz tones (0.82) it was clear that a ceiling had been reached. West et al. (2000) also had observers judge the brightness of lights on the same standard scale, in this case reproducing Stevens’ standard finding of an exponent very near 0.3 for brightness, ½ of his recommended exponent for loudness, and doing so for *each* of their eight participants separately. These results counter the possible criticism that training participants to use a standardized response scale could distort accepted psychophysical findings. At least for the continua tested by West et al. (2000), as for the experiments of Marks et al. (1995), who found a similar preservation of binaural additivity using trained participants, this was not the case.

In another study, West and Ward (1998; West, 1996) had participants scale their happiness at winning various amounts of money ($50 to $1,000,000) in a lottery, using the same standard scale to which they had been trained. Intriguingly, while they remained calibrated to the standard exponent for loudness of 1000 Hz tones (mean = 0.57; range 0.48–0.61), participants’ exponents for money-induced happiness varied widely from the mean of 0.24 (range 0.06–0.39). These exponents represent extreme individual differences; for example, to double the happiness of the 0.06-exponent-participant would require 100,000 times as much money, whereas to double the happiness of the 0.39 exponent person would require only about six times as much money. This result demonstrates that CS does not suppress individual differences for continua where such differences are expected, while at the same time constraining participants to use a common scale for expressing those differences.

West et al. (2000) also speculated that CS could be used to achieve precise quantitative reproduction of results across experiments in different laboratories. A simple model (e.g., Curtis et al., 1968; Marks, 1991; West et al., 2000) can be used to see why this is plausible but is not guaranteed by the West et al. (2000) results on reduction of inter-individual variability. The psychophysical function is generally written as *R = f(S) = aS^m^*, where *R* is the average response to stimulus magnitude *S*, and the function *f* is a power function with unit *a* and exponent *m*. In the model, however, the relationship, *f*, between *R* and *S* is decomposed into two functions, *f = C(P), P* for the perceptual transform of the stimulus, and *C* for the cognitive transform of the percept, thus *R = C[P(S)]*. The goal of psychophysical scaling is arguably (e.g., Stevens, 1975) to discover the function *P*, the psychophysical function that represents the relationship between stimulus magnitude and sensation magnitude. But fitting a simple power function to response and stimulus magnitudes confounds the *P* and *C* transforms; the fitted power function reflects *P* alone only if *C* is the identity function, and its exponent is the sought-after one only if *C* is linear. In this model *C* is cognitively penetrable (Fodor, 1983) whereas *P* is not; *P* reflects sensory and perceptual processing occurring relatively early and automatically in the sensory system. The function *C*, on the other hand, represents how the participant chooses to report his or her sensation magnitude, and is influenced both by internal factors such as understanding of the instructions of the experiment, previous experience with the reporting variable(s), previous experience with the continuum to be judged, experience with previous stimuli and responses, discrimination problems caused by sensory and memory noise (one possible influence of stimulus range, see Ward et al., 1996 and references therein), various response biases (e.g., Poulton, 1989), and the desire (or lack thereof) of the participant to please the experimenter, and by external factors such as the actual instructions given (including especially the response continuum chosen by the experimenter and the manner in which it is to be used for reporting), the manner in which those instructions are given (including non-verbal cues), feedback as to appropriateness of the participant’s performance (cf. cartoon in Frontispiece of Poulton, 1989, depicting S.S. Stevens giving feedback to a participant), and the experimental context including equipment, general environment (soundproof chamber or lab room, outside noise, etc.), and social factors such as gender, authority, etc. of the experimenter.

The internal factors influencing *C* obviously would contribute to inter- and intra-participant variability in scaling results, and West et al. (2000) showed that CS substantially controlled the influence of those internal factors. West et al. (2000) did not, however, address the extent to which CS controlled any of the external factors, taking place as it did in only a single laboratory and run by only a small group of experimenters from that lab. The external factors affecting *C* undoubtedly are responsible for much of the inter-laboratory and inter-experiment variability mentioned earlier. Rather than trying to model or counterbalance for all of the multifarious factors just listed, both internal and external, CS presents a standard context, set of instructions, training, and experience that should control the external factors as well as the internal factors, rendering *C* as nearly as possible the same across time, individuals, experiments and laboratories, and therefore rendering more precise replicability of scaling results across laboratories and across experiments. This paper represents a first attempt to demonstrate the efficacy of CS in controlling the external factors influencing *C*.

## Materials and Methods

### Participants

In order to investigate reproducibility across laboratories, we collaborated in replicating experiments in our four different laboratories using participants obtained locally: University of British Columbia (UBC; total *n* = 38), McMaster University (total *n* = 33), and Carleton University (total *n* = 18) in Canada and Tokyo Metropolitan University (total *n* = 15) in Japan (administered in Japanese). The paradigm was approved by the ethics committees at each institution and we obtained informed consent from our participants in accordance with the procedures established by the ethical review committees at each institution.

### Apparatus and Procedure

Each laboratory used the same CS procedure and graphical user interface (see below) but different sound generation, presentation, and calibration apparatus. A standard SoundBlaster sound card, a custom artificial ear, a Quest Technologies Model 2700 Precision Sound Level Meter, and Kenwood KPM-510 headphones were used in all experiments at UBC; a Tucker–Davis sound generator (RP2) and programmable attenuator (PA5), a Tucker–Davis artificial ear, and Sennheiser HD-200 headphones were used at McMaster; a SoundMax Integrated Digital Audio sound card from Analog Devices Inc., a Type 4153 Bruel & Kaer artificial ear, a Type 2260 Bruel & Kaer Modular Precision Sound Analyzer, and ATC-HA7USB Audio-Technica USB Digital Headphones were used at Tokyo Metropolitan; a VIA AC’97 generic motherboard sound card, a custom plaster-of-Paris artificial ear, a Sper Scientific Ltd. Item 840015C sound level meter, and Sennheiser DH 280 Pro 64 ohm headphones were used at Carleton.

**FIGURE 1:**
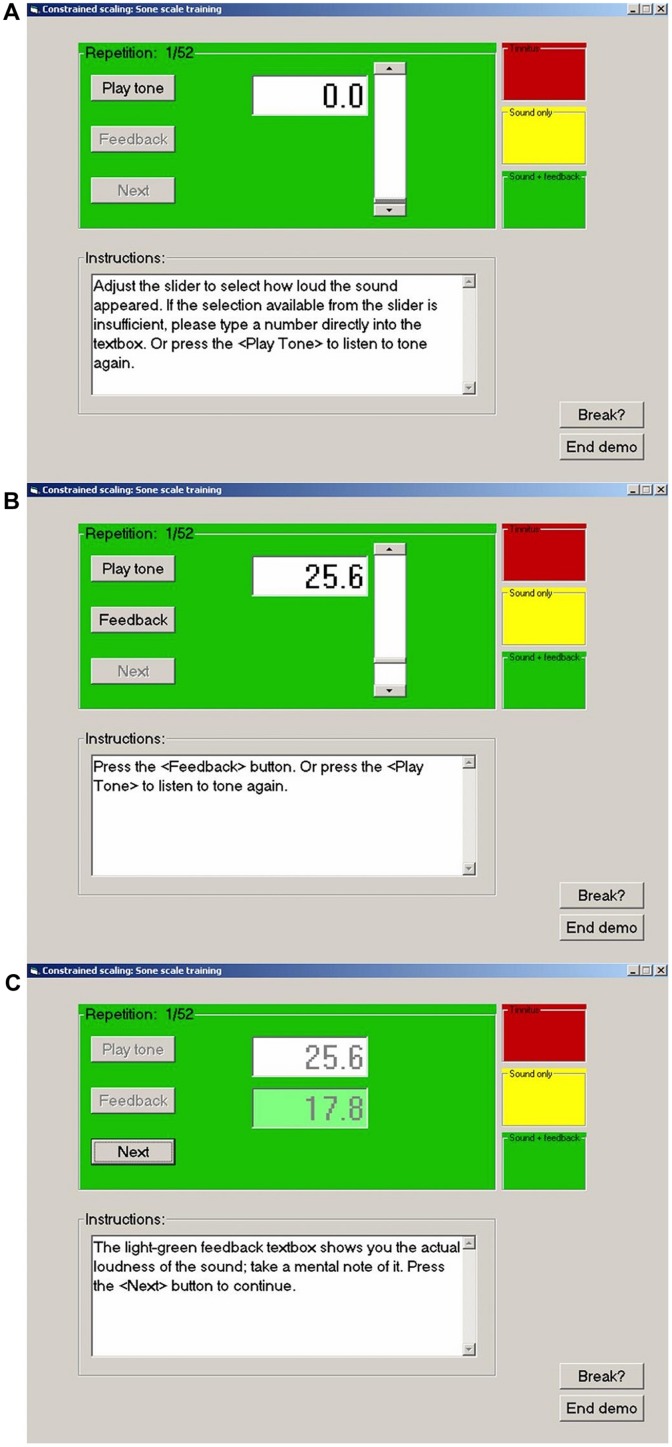
**Examples of the computer graphical user interface used in the present experiments. (A)** Before presentation of a tone. **(B)** Subject has presented the tone and entered a response (25.6). **(C)** Subject has received feedback (17.8) and is ready to proceed to the next stimulus. For no feedback trials the feedback button is not activated and the feedback box remains empty.

The CS procedure used by each laboratory to train participants at loudness estimation on the modified sone scale is summarized in **Figure 1**. The graphical user interface seen by the participant is illustrated for a single trial. In step (a) participants pressed a button to play a 1000 Hz tone of 1 s duration. In step (b) they used a slider to select a number estimating the loudness of the tone on the trained scale (25.6 in this example). An option was provided to hear the tone again before confirming their estimate. In step (c) feedback was given for the actual loudness of the tone on the trained scale. Participants were asked to make a mental note of this value and to proceed to the next trial where a tone of different level was presented. The experiments reported herein for each laboratory commenced by training participants to estimate the loudness of a 1000 Hz tone on the modified sone scale used by West et al. (2000), *R* = 16.6 *P*^0.60^, using this procedure.

**FIGURE 2:**
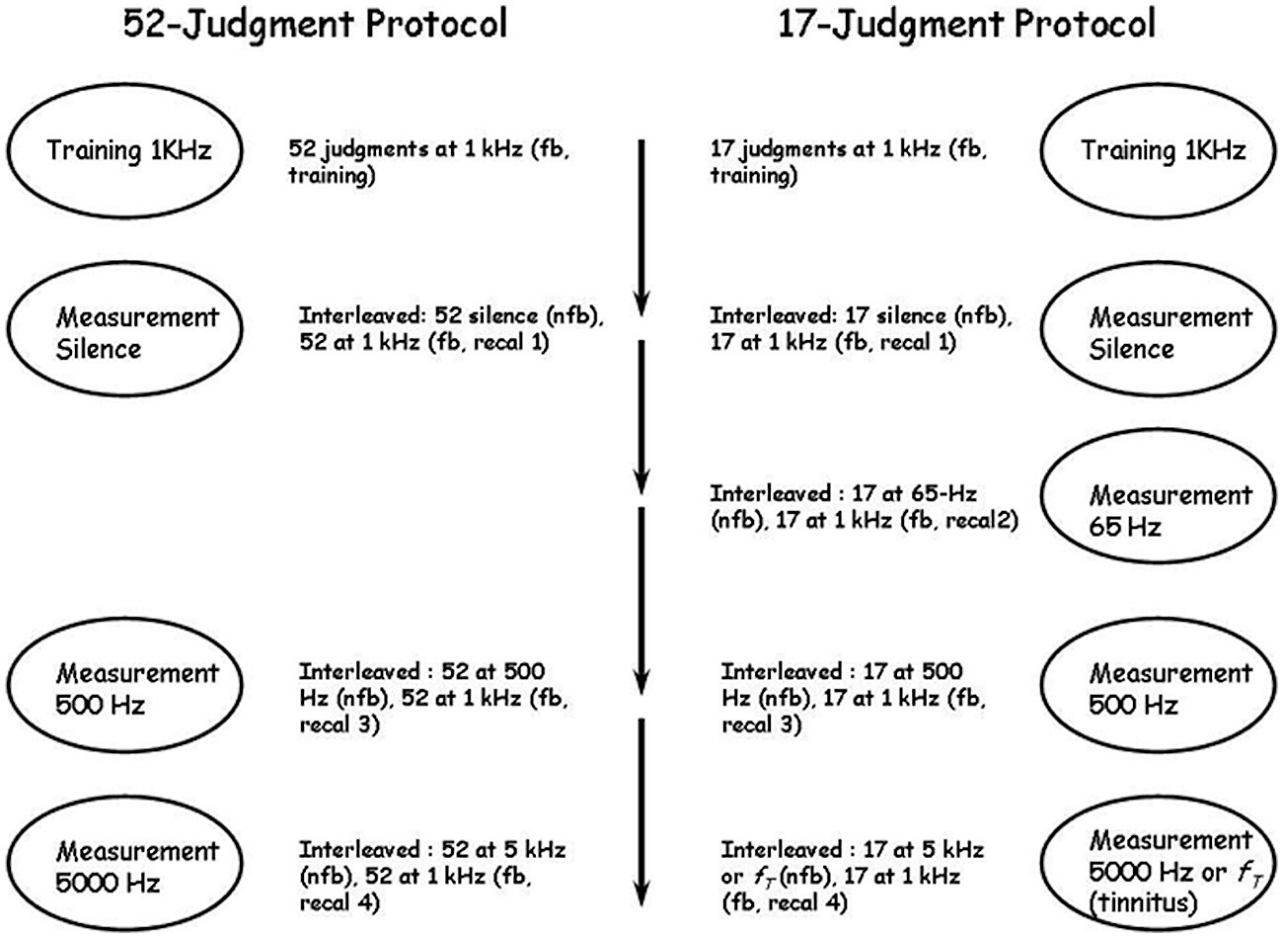
**The 52- and 17-judgment protocols.** Fb, feedback; nfb, no feedback; recal, recalibration; f_*T*_, tinnitus frequency. In the 52-judgment protocol there were three series of measurements following the first training series and separated by short breaks; in the 17-judgment protocol there were four series of measurements following the first training series and also separated by short breaks. The ovals indicate what was measured in each series and the material beside each oval indicates the details of the series. In all measurement series recalibration trials at 1 kHz with feedback were interleaved with measurement trials on the continuum to be measured.

The stages of two different experimental protocols are summarized in **Figure 2**. In both protocols observers first learned the standard scale using 1000 Hz tones and then produced judgments of pure tones at several different, untrained, frequencies, including 500 Hz, 5000 Hz, silence, and in one protocol 65 Hz. Judgments of silence were universally rated “0” and are not discussed further except where indicated for tinnitus sufferers reported by Ward and Baumann (2009). We also manipulated the number of training, judgment and calibration stimuli in an effort to discover the limits of the technique. In the first experimental protocol [run in only two of the labs and designated UBC-52 (*n* = 10) and McM-52 (*n* = 18)], observers made 52 training judgments of 1000 Hz tones with feedback, then 52 judgments of the same stimuli with feedback (termed “recalibration runs” and numbered according to their place in the overall protocol; this would be “Recal 1”; see **Figure 2**) interleaved with 52 judgments of “silence” without feedback, for a total of 104 judgments of 1000 Hz with feedback before judging pure tones of other frequencies. They then made 52 judgments of 500 Hz tones without feedback interleaved with 52 judgments of 1000 Hz tones with feedback (Recal 3), followed by 52 judgments of 5000 Hz tones without feedback interleaved with 52 judgments of 1000 Hz tones with feedback (Recal 4). In each case, the levels of the 52 stimuli ranged from 40 to 90 dB in 1-dB steps and one trial with no stimulus was also included. All stimuli were presented *only one time each* in a different shuﬄed order in each run. The entire experiment was completed in approximately 1 h. In the second experimental protocol [run in all four labs, and designated UBC-17 (*n* = 15), McM-17 (*n* = 15), Carl-17 (*n* = 18) and TMU-17 (*n* = 15)], only 17 judgments were made in each set, instead of 52, and in addition 17 judgments of 65 Hz tones without feedback were made interleaved with 17 judgments of 1000 Hz tones with feedback (Recal 2). The 17 stimuli in this second protocol consisted of levels from 40 to 88 dB in 3-dB steps (the no-stimulus trial was omitted), and were again presented one time each in a shuﬄed order in each run. The entire experiment was completed in approximately ½ h. This 17-judgment protocol was included specifically to determine whether a very brief protocol, designed to be of use in the clinic with participants who were likely to have physical health challenges and who could not complete long sessions with many judgments, could approach in precision the results of the longer protocols employed by West et al. (2000) and the 52-judgment protocols of the present study.

We also ran in this second protocol, at UBC, a group of participants [UBC-17t (*n* = 14)] all of whom were suffering from tinnitus (ringing in the ears). Two additional tinnitus participants are not included in this group because they failed to learn the scale because of profound hearing loss. Tinnitus participants judged sounds at their measured tinnitus frequency instead of at 5000 Hz (data not reported here) but performed all other runs in the same way as the other participants. These data, and their implications for clinical use of CS, are described more extensively elsewhere (Ward and Baumann, 2009). The mean results for 1000, 500, and 65 Hz are included here both because tinnitus sufferers are a special population for whom such measurement is especially useful, and also to increase the number of separate replications of the protocol.

### Analysis of Data

It was necessary to find a way to characterize the existing level of quantitative reproducibility of scaling results across experiments and laboratories. After considering many alternatives, we decided to use the two indicators used by West et al. (2000): the SD of a set of exponents divided by the mean of that set (*SD/M*, the coefficient of variation), and the ratio of the highest to the lowest exponent in the set [*High/Low (H/L)*]. West et al. (2000) showed that CS reduced these indicators, calculated across individuals, from values ranging from 0.19 to 0.45 for *SD/M* and 1.6 to 6.0 for *H/L* (from the literature summarized in their **Table 1**) to 0.045 to 0.100 for *SD/M* and 1.2 to 1.4 for *H/L* (for experiments where observers were trained to the sone scale) across all of their experiments and the various psychological continua they used. To obtain such values for average exponents from groups of participants run in different experiments and different laboratories, we selected 26 average exponents reported by various authors and summarized in Table 1 of Marks (1974b) for magnitude estimations of 1000 Hz tones, and 13 additional average 1000-Hz exponents from magnitude estimation experiments reported in various papers by one of the authors (Ward, references supplied on request, including two control experiments reported by West et al. (2000) using conventional techniques, and all run in the same laboratory). For these 39 exponents, the value of *SD/M* was 0.12/0.54 = 0.22 and that of *H/L* was 0.93/0.30 = 3.10. In this paper we compare these values, and some subsets of them, to the results of a range of CS exponents obtained in several experiments run in four different laboratories.

**Table 1:** Average across observers of exponents (*Mean m*) and *SD/M* values for judgments of the loudness of 1000 Hz pure tones during several runs with feedback.

Study	Training	Recal 1	Recal 2	Recal 3	Recal 4
**UBC-52**					
*Mean m*	0.55	0.53	N/A	0.52	0.49
*SD/M*	0.06	0.08	N/A	0.06	0.12
**McM-52**					
*Mean m*	0.58	0.56	N/A	0.55	0.51
*SD/M*	0.14	0.08	N/A	0.12	0.10
**UBC-17**					
*Mean m*	0.57	0.55	0.52	0.51	0.47
*SD/M*	0.21	0.18	0.21	0.17	0.21
**McM-17**					
*Mean m*	0.56	0.54	0.48	0.48	0.47
*SD/M*	0.21	0.19	0.22	0.16	0.19
**TMU-17**					
*Mean m*	0.51	0.52	0.48	0.50	0.49
*SD/M*	0.15	0.11	0.14	0.16	0.11
**Carl-17**					
*Mean m*	0.54	0.51	0.49	0.49	0.45
*SD/M*	0.18	0.15	0.22	0.26	0.25
**UBC-17t**					
*Mean m*	0.59	0.52	0.51	0.51	0.52
*SD/M*	0.30	0.16	0.17	0.15	0.14
**West et al. (2000)-100**					
*Mean m*	0.59	0.54	0.54^∗^	0.55	N/A
*SD/M*	0.03	0.04	0.07^∗^	0.07	N/A
**West et al. (2000)-50**					
*Mean m*	0.56	0.56	N/A	N/A	N/A
*SD/M*	0.11	0.08	N/A	N/A	N/A

*^∗^no feedback run with 1000 Hz tones.*

Power functions were fitted to the *individual judgments* using linear regression on the logarithms of sound pressures and responses. Responses on the no-stimulus trial (52-trial protocol, nearly universally rated “0”), and any other responses of “0” were not included in the curve fitting. Exponents, *m*, in

(1) $$R=aP^{m}$$

where *R* is the response and *P* is the sound pressure of the stimulus were estimated from:

(2) $$\log R=\hat{m}\log P+\log\hat{a}+error,$$

where is 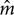 the estimate of *m*, â is the estimate of *a, error* is the fitting error, and the first three terms of Eq. 2 are the log-transformed version of Equation 1. West et al. (2000) discussed the problem of fitting power functions to such data, including how to estimate the effect of statistical regression on the estimate of the exponent caused by fluctuations in subjects’ judgments (*error* in Eq. 2). This effect can be characterized by 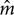 = mr_RP_ where *r_RP_* is the correlation coefficient between an observer’s responses and the sound pressures of the stimuli to which they were made (personal communication from Rule, 1998, cited and explained further in West et al., 2000). Thus, exponents estimated from a set of judgments are always smaller than the exponent that would be measured if there were no “error” in subjects’ judgments unless *r_RP_* = 1, which is possible but in practice never the case.

Random fluctuations in subjects’ judgments are probably inevitable in psychophysical scaling, as everywhere else, and usually we wish to minimize their impact on our conclusions. They must be dealt with carefully in CS, in particular, because *we fit power functions to individual judgments* rather than to median or mean judgments. This should amplify regression effects because averaging or taking medians, as typically done, allows some of the fluctuations to cancel or to be swamped. Remarkably, in West et al. (2000) the regression effect explained most of the small departures of estimated exponents from training exponents for standard stimuli, and seemed to cause little concern for non-standard stimuli. If the effect becomes too large, however, the estimated exponents will not be accurate enough for scientific use. In the present experiments, we judged that this regression effect would be too large for accurate estimation of the exponent if *r_RP_* was less than about 0.82 (*r_RP_*^2^ < 0.67), where stimulus variation explains about 2/3 of the variance in responses. This never occurred for 1000-Hz runs with feedback in the 52-stimulus protocol, but did occur for a few 1000-Hz runs with feedback in the 17-trial-protocol experiments and somewhat more often for the no-feedback 500, 5000, or 65 Hz stimulus sets. Clearly subjects had a harder time judging stimuli from these novel frequencies, and 17 trials of training on the standard scale were not enough for them always to be able to use it reliably to characterize their sensations arising from non-standard stimuli. Data presented in **Table 1** (see Section “Results” for this and other tables) are based on all 1000-Hz runs, regardless of *r_RP_*^2^, but we do not include in **Table 2** the data from any run in which *r_RP_*^2^ < 0.67. **Table 2** also displays the numbers of subjects meeting this criterion for each, corresponding, exponent listed in the table. These proportions constitute data about CS in themselves and are discussed in the Section “Results.”

**Table 2:** Average across observers of exponents (*Mean m*) and *SD/M* values for judgments of the loudness of 500, 5000, and 65 Hz pure tones without feedback.

Study	65 Hz	N 65 Hz	500 Hz	N 500 Hz	5000 Hz	N 5000 Hz
**UBC-52**						
*Mean m*	N/A	N/A	0.51	9/10	0.54	9/10
*SD/M*	N/A		0.12		0.15	
**McM-52**						
*Mean m*	N/A	N/A	0.54	16/17	0.57	16/17
*SD/M*	N/A		0.12		0.20	
**UBC-17**						
*Mean m*	1.02	10/15	0.49	14/15	0.50	15/15
*SD/M*	0.25		0.18		0.24	
**McM-17**						
*Mean m*	0.97	13/15	0.48	13/15	0.47	10/15
*SD/M*	0.45		0.16		0.14	
**TMU-17**						
*Mean m*	0.74	14/15	0.53	12/15	0.47	13/15
*SD/M*	0.31		0.25		0.27	
**Carl-17**						
*Mean m*	0.98	15/18	0.45	16/18	0.48	10/18
*SD/M*	0.27		0.30		0.28	
**UBC-17t**					**f(tinn)**	
*Mean m*	0.89	11/14	0.46	14/14	0.80	11/14
*SD/M*	0.40		0.28		0.50	
**West et al. (2000)-100^∗^**						
*Mean m*	0.70	6/6	N/A	N/A	N/A	N/A
*SD/M*	0.11		N/A		N/A	
**West et al. (2000)-50^∗^**						
*Mean m*	0.67	7/7	N/A	N/A	N/A	N/A
*SD/M*	0.09		N/A		N/A	

*Cell entries based on numbers of valid subjects/total number of subjects indicated in neighboring columns (N).*

*^∗^Uncorrected for near threshold curvature.*

Our practice here is justified by the following reasoning. There is, in principle, a trade-off between excluding runs that do not meet the *r_RP_* criterion, which tends to make the data appear better than they are, and using uncorrected exponents from the runs we do include, none of which have *r_RP_*^2^ = 1.0, which tends to make the data appear worse than they could be if a regression-related correction were applied. For the 17-stimulus protocol in particular, however, the estimation of the power function exponent is also vulnerable to an under-sampling error, because each response is only one of several that reasonably could have been given to that particular stimulus, albeit this set is much smaller in CS than in conventional scaling. Thus, alternative responses by a subject to only a few of the 17 stimuli in the set could alter the estimated exponent considerably while leaving *r_RP_*^2^ nearly unchanged. This vulnerability cannot be compensated for by a simple correction based on the regression, since the regression does not reflect it. Thus, we decided to exclude the few more variable data sets and to report uncorrected exponents rather than to correct all exponents for the regression effect. It should be mentioned here that this problem is a general one in curve fitting and has not yet been adequately addressed that we know of, although resampling and/or Bayesian methods (e.g., Dixon and O’Reilly, 1999) may one day provide a better solution than we have achieved. Until this happens, in practice, *r_RP_*^2^ for the more difficult continua should be estimated every several trials and the run terminated only when it exceeds the minimum criterion.

## Results and Discussion

**FIGURE 3:**
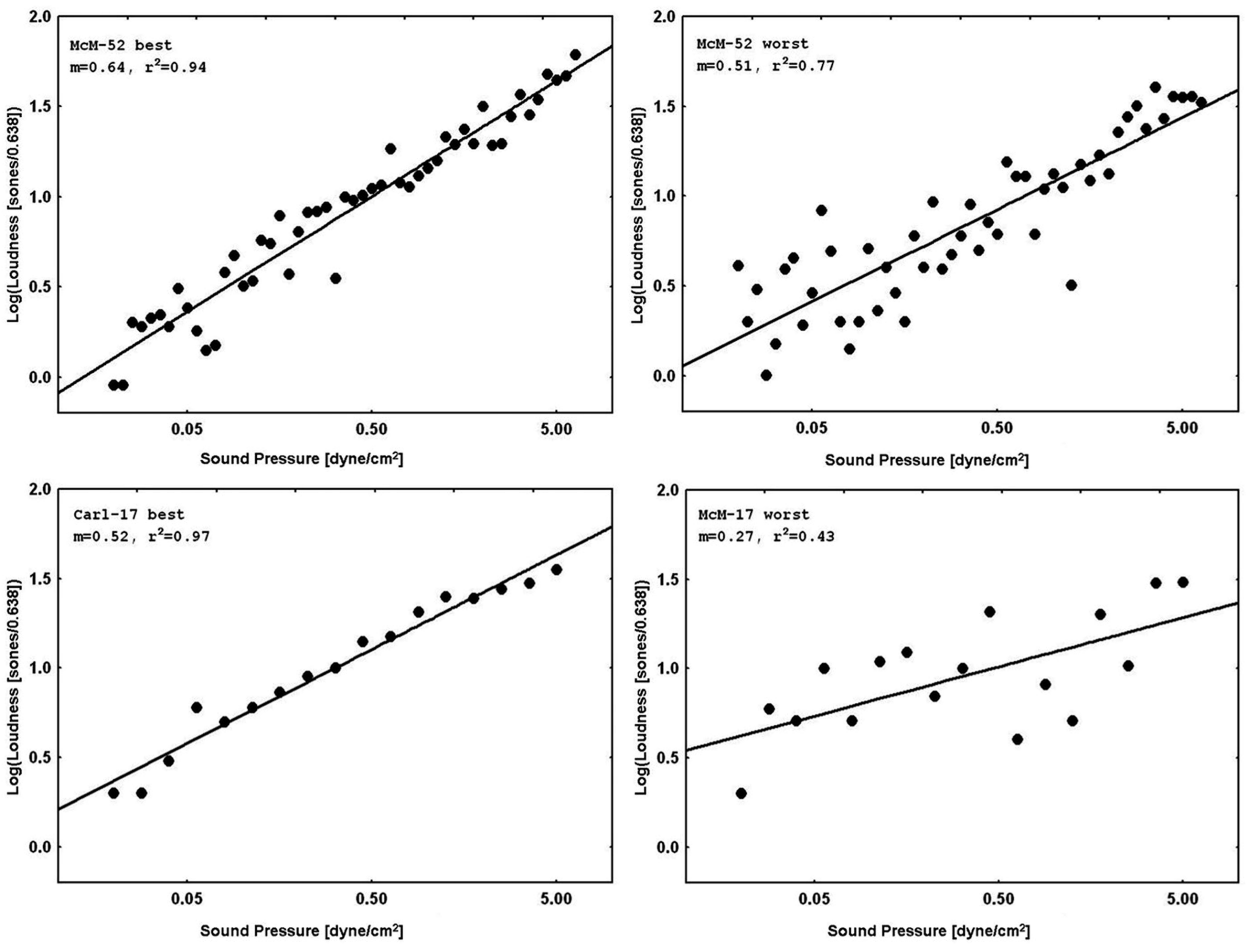
**Representative psychophysical functions of “best” and “worst” observers for judgments of loudness of 1000-Hz pure tones *with* feedback from 52-stimulus and 17-stimulus protocols run in the four different laboratories.** These functions are from the first recalibration runs (interleaved with judgments of silence).

**FIGURE 4:**
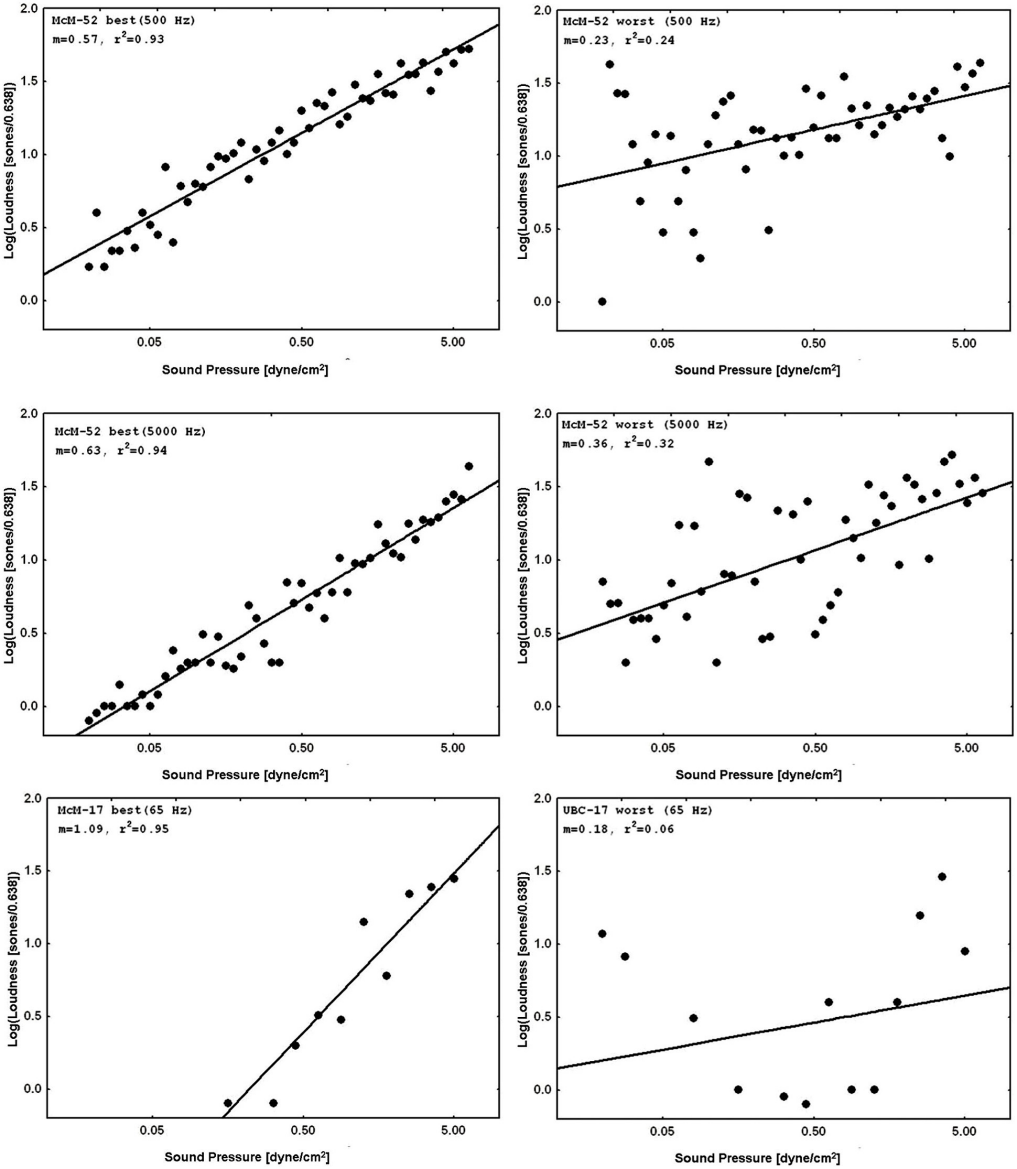
**Representative psychophysical functions of “best” and “worst” observers for judgments of loudness of 65, 500, or 5000 Hz pure tones from 52-stimulus and 17-stimulus protocols run in the four different laboratories.** These functions are from runs in which judgments of tones at the indicated frequency *without* feedback were interleaved with judgments of 1000 Hz tones with feedback.

**Figure 3** displays representative psychophysical functions from the “best” and “worst” observers (in terms of *r_RP_*^2^) across all four laboratories from the present experiments. These are from the first recalibration run at 1000 Hz with feedback (interleaved with judgments of silence for the normal subjects, and with judgments of tinnitus magnitude for the tinnitus sufferers) for the 52-stimulus and the 17-stimulus protocols separately. **Figure 4** does the same for the 65, 500, and 5000 Hz data. It should be stressed that *individual responses to individual stimuli* are plotted in **Figure 3** and in **Figure 4**, in contrast to usual psychophysical functions that, even when plotted for individual observers, consist of points based on from several (around 10 is a typical minimum) to many (sometimes over 50) judgments per stimulus. This renders the present functions even more impressive because the well-known variability of responses to repeated presentations of the same stimulus has *not* been averaged out (there *were* no repeats of the same stimulus, of course, in these functions). The functions in **Figure 3** and in **Figure 4** are very comparable to those reported by West et al. (2000). The worst observer for the 17-stimulus protocol and several others across the various experiments with this protocol, however, as mentioned earlier, had *r_RP_*^2^ values lower than our rule-of-thumb criterion of 0.67 for at least one run. Overall, about half of the observers in the 17-stimulus experiments had at least one 1000-Hz run (from among four or five that they completed), usually a later recalibration run, that fell below our criterion. And several observers in each experiment had *r_RP_*^2^ < 0.67 for at least one run for the other frequencies, represented in the “worst” cases for these frequencies. Clearly for these observers on those runs 17 trials were not enough for a reliable estimate of the power function exponent, particularly for novel stimuli.

**Table 1** summarizes the data from the 1000 Hz runs, all with feedback, from the seven current experiments plus two from West et al. (2000; their experiments 1A, designated West et al. (2000)-100, and 1B, designated West et al. (2000)-50, with different subjects and different protocols, in particular 100 and 50 stimuli, respectively, of each type in each run). All of the current data are very comparable to those of West et al. (2000), both to their 1000 Hz runs with feedback (recalibration runs) and also to the 1000 Hz run without feedback of West et al. (2000)-50, which had identical exponents and comparable *SD/M* and *H/L* statistics across individuals to their other runs. Most important, it is easy to see that the exponent values themselves are quantitatively very similar across laboratories, as are the *SD/M* values. There is, as might be expected, more variability across individuals in the 17-stimulus experiments: the *SD/M* values are about the same as the best of the standard magnitude estimation experiments surveyed by West et al. (2000), although much better than the average of those. Clearly there is a cost of estimating power function exponents from psychophysical functions based on individual judgments using so few trials, both in terms of the regression effect discussed in the Method section, and also possibly in subjects’ learning of the standard scale. West et al. (2000)-100 had observers perform 1000 judgments in total in their Experiment 1A [West et al. (2000)-100], of which 800 were at 1000 Hz (600 with feedback), and 200 at 65 Hz. In contrast, in the 17-stimulus protocol, our observers performed only a *total* of 85 judgments of 1000 Hz tones with feedback. Thus, their *total* experience was not even as great as the first training run in West et al. (2000)’s Experiment 1A! It is remarkable that this degree of quantitative precision can be achieved with so few trials using the CS technique. Given that each trial consumes about 10 s, even during the training run, this represents a huge savings in the time required to assess sensory function in this way. Only about 3 min per run is required – in an hour many different measurements can be made.

We can summarize the effect of CS on across experiment and across-laboratory variability by comparing the *SD/M* and *H/L* statistics for these replications using CS with those for CME mentioned earlier. Over the 39 average CS exponents displayed in **Table 1**, *SD/M* = 0.03/0.52 = 0.06, and *High/Low* = 0.59/0.47 = 1.60, clearly an improvement over the same statistics for the conventional technique (0.22 and 3.10, respectively, as mentioned above). Similar statistics can be obtained for any individual column of **Table 1**, which is perhaps more comparable to the 39 exponents from separate experiments in the literature estimated using an unconstrained technique. For example, for the first recalibration, for which nine exponents for different groups of subjects across four different labs are available (Recal 1 column in **Table 1**), *SD/M* = 0.02/0.54 = 0.04, and *High/Low* = 0.56/0.51 = 1.10. These statistics are even better than for the entire group of 39 exponents in **Table 1**.

The difference between statistics for a single recalibration versus taking all of the **Table 1** exponents together possibly arises from fatigue or boredom effects increasing judgment variability, and thus lowering the estimated exponent values via the regression effect described in the Method section, as the experiments progressed. Although we have no independent assessments of fatigue or boredom, this interpretation is supported by the observation that exponent values generally decreased across recalibrations, with averages across the experiments of 0.56, 0.54, 0.50, 0.51, 0.49 for training and recalibrations one through 4, respectively. This difference in exponent values is statistically reliable. Because of protocol differences we ran two separate repeated measures ANOVAs (using the MANOVA approach to avoid bias arising from sphericity violations, and α = 0.05 for statistical significance, here and throughout) for the 52-stimulus and 17-stimulus protocols. In the first of these we examined the factors Experiment (UBC-52, McM-52) and Recalibration (Recal 1, Recal 3, and Recal 4). We did not consider the training run and these groups had no 65 Hz runs and so no “Recal 2” run either. This analysis showed a significant main effect of Recalibration [*F*(2,50) = 11.13, *p* < 0.001, η^2^ = 0.138] but no effect of Experiment and no interaction. The second analysis, for the 17-stimulus protocol, had factors Experiment (UBC-17, McM-17, TMU-17, Carl-17, UBC-17t) and Recalibration (Recal 1, Recal 2, Recal 3, Recal 4). This analysis also revealed a significant, although slightly smaller, main effect of Recalibration [*F*(3,159) = 2.87, *p* < 0.04, η^2^ = 0.025] but no effect of Experiment and no interaction. Recall that the 17-judgment protocol required much less time than the 52-judgment protocol, even with an additional measurement series (1/2 h versus 1 h), and thus was considerably more efficient. Nonetheless, some decline in precision on the standard 1-KHz continuum was still present in the 17-judgment protocol.

The increase in judgment variability leading to the general decrease in recalibration exponent values for the same stimuli across repeated runs could also contribute to somewhat lower values for exponents for non-standard continua run in the later parts of the experiment. Again, a correction for this effect could be attempted, such as multiplying each later, no-feedback exponent by the ratio of the training exponent to the recalibration exponent (both at 1000 Hz with feedback), but we did not do that here. Instead we compared each such exponent with its particular recalibration exponent obtained from the same run of interleaved trials. Thus, the results discussed next represent a conservative test of the method but with some control for the decline in recalibration exponent across runs in the same experiment.

Although CS clearly improves across-experiment consistency on the training continuum with feedback, this is not a very stringent test of the method’s usefulness to scale stimuli on non-standard continua. West et al. (2000) showed that a similar quantitative precision across individual subjects was attainable for non-standard continua judged without feedback, including other sound frequencies and also brightness of lights. A similarly stringent test in the present context would be to address the reproducibility of the judgments of off-training-frequency stimuli across laboratories and experiments. **Figure 4** and **Table 2** present the data for judgments of 65, 500, and 5000 Hz pure tones. The single-judgment-per-stimulus psychophysical functions plotted in **Figure 4** show that the results for judgments of the non-standard frequency stimuli without feedback are generally comparable to those of the standard stimuli with feedback (cf. **Figure 3**). **Table 2** presents a similar but slightly more complicated summary to that of **Table 1**, also remembering that these average exponents are only from runs that met our accuracy criterion. (As mentioned earlier, in practice subjects could be run until they met the criterion for all runs – here we decided not to do this). **Table 2** also presents the numbers of subjects who had valid runs for each experiment and sound frequency; the average exponents and *SD/M* values presented in **Table 2** are based on the number of subjects indicated in the adjacent cells of that table.

Considering first only the average exponents in **Table 2**, we do find excellent agreement across laboratories in the exponents for 500 and 5000 Hz, although the *SD/M* across observers is typically somewhat larger than for the 1000 Hz training frequency tones. The overall average exponents for 500 and 5000 Hz are expected from previous results (e.g., Ward, 1990) to be close to those for 1000 Hz and they are. Over the 7 (respectively 6) replications of each set of judgments across laboratories and protocols (excluding UBC-17t from the 5000 Hz figures because those subjects did not judge 5000 Hz), the *SD/M* and *High/Low* statistics are, respectively, 0.03/0.50 = 0.06 and 0.54/0.45 = 1.20 for 500 Hz, and 0.04/0.51 = 0.08 and 0.57/0.47 = 1.21 for 5000 Hz. These numbers represent excellent reproducibility across laboratories, comparable to that achieved across individuals in the recalibration runs with feedback (viz. *SD/M* values reported in **Table 1**) and somewhat better than the reproducibility across individuals for 500 and 5000 Hz (**Table 2**). Moreover, not many runs were excluded from these averages; from **Table 2** we see that the average proportion of subjects included was around 0.86; this usually amounted only to one or two excluded runs although in two cases for 5000 Hz, McM-17 (5 of 15 excluded) and Carl-17 (8 of 18 excluded), a larger number of subjects did not meet our criterion.

We did several analyses of variance to examine these impressions more closely. In these analyses we used only exponents estimated from psychophysical functions that met our criterion in order to limit the influence of the regression bias. Thus, we also excluded a few exponents from the 1000 Hz data, although over 92% of the 1000 Hz runs met our criterion and thus were usable. This practice did result in some subjects’ data not being included in every analysis, however, so the results should be interpreted with this in mind.

A mixed between (UBC-52, McM-52, UBC-17, McM-17, TMU-17, Carl-17)-within (500 Hz, 1000 Hz_recal500,_ 5000 Hz, 1000 Hz_recal5000_) groups ANOVA revealed neither any significant main effect nor any interaction. Thus, these experiments gave rise both to the expected result when comparing directly estimated exponents for these three frequencies and also to the same results in the four labs and two different protocols in which the experiments were run. A planned contrast between the 52-stimulus and the 17-stimulus protocols indicated that the exponents for 500 and 5000 Hz were higher for the 52-stimulus protocol [univariate *F*(1,56) = 8.19, *p* = 0.006], a result explained by the inverse relation between the size of the regression effect and the number of stimuli judged, resulting in a smaller regression effect for the 52-stimulus protocol. Interestingly, the 52-stimulus recalibration (1000 Hz) exponents were the same as the 17-stimulus ones [univariate *F*(1,56) = 0.74, *p* = 0.39]. Apparently feedback and more practice on the standard stimuli were able to lessen the impact of the regression effect in the recalibration runs, especially for the 17-stimulus protocol. Given that the average exponent difference between 52-stimulus and 17-stimulus protocols was only approximately 0.04 and 0.08 for 500 and 5000 Hz, respectively, this indicates that comparisons should not be made across protocols with different numbers of stimuli without accounting for the fact that the regression effect might differ with number of stimuli used in the psychophysical functions that assess the test continua. In general, however, this result is encouraging in the sense that never before has it been possible to discriminate exponent differences across experiments with this degree of precision. S.S. Stevens dreamed of the day when one decimal point precision would be routine in psychophysical scaling (personal communication to Ward in 1969). We believe that these results indicate that we are approaching that status with CS.

Similar analyses were also done for the 65 Hz judgments separately because the exponent for 65 Hz is expected to be substantially greater than that for 1000 Hz (e.g., Marks, 1974a; Ward, 1990; West et al., 2000). These analyses were restricted to the 17-stimulus protocol because this frequency was not judged in the 52-stimulus protocol experiments. Over the five different experiments *SD/M* = 0.12 and *H/L* = 1.38. Although somewhat higher than for the other test frequencies these are still quite good values, and still drastically lower than achievable with conventional direct scaling. Here the mixed between (UBC-17, McM-17, TMU-17, Carl-17, UBC-17t) – within (1000 Hz_recal65_, 65 Hz) analysis revealed a significant main effect of Frequency as expected [*F*(1,52) = 98.64, *p* << 0.001, η^2^ = 0.448] and no interaction with the Experiment factor. Thus, all of the experiments replicated the typical finding that the exponent for 65 Hz is significantly greater than that for 1000 Hz. Interestingly, however, there was also a marginal main effect of Experiment [*F*(4,52) = 2.46, *p* = 0.06, η^2^ = 0.047). Inspection of the means in **Table 2** reveals that the TMU-17 experiment yielded a somewhat lower exponent for 65 Hz than did the others. Moreover, the average exponents (overall average 0.92) displayed for the present experiments in **Table 2** are significantly larger than those reported by West et al. (2000), viz. 0.70 and 0.67 [*t*(51) = 5.00, *p* << 0.001 and *t*(51) = 5.69, *p* << 0.001, respectively], but that of the TMU-17 experiment alone is not [*t*(14) = 0.70, *p* = 0.49 and *t*(14) = 1.20, *p* = 0.25]. The reason for the quantitative disagreement between the present results and those of West et al. (2000), and that between TMU-17 and the other 17-stimulus experiments, is not clear. It may arise from the fact that observers in the 17-stimulus protocol had trouble judging these stimuli at all and gave very low or zero ratings to many of the lower sound pressure levels. This would tend to increase log-log regression slopes, and thus increase estimated exponents based on those judgments. Moreover, the *SD/M* values in our 65-Hz 17-stimulus experiments are the highest we have ever seen using CS, and this is after excluding several runs from the averages because of *r_RP_*^2^ values lower than 0.67. Perhaps the small number of judgment trials, and the concomitant lack of practice judging these hard to hear stimuli (thresholds typically 40 dB or higher), reduced the efficacy of the technique. Indeed two of the UBC-17 observers who were excluded from this analysis had non-monotonic psychophysical functions for the 65 Hz stimuli, indicating that they were simply guessing the appropriate response. These observers may have had an undiagnosed low-frequency hearing loss. The fact that the recalibration judgments of 1000 Hz stimuli interleaved with the 65 Hz judgments for these observers remained normal, as did their other psychophysical functions, indicates that the problem was only with the 65 Hz stimuli. As mentioned earlier, West et al. (2000)’s observers performed 200 judgments or 50 judgments of the 65 Hz stimuli; perhaps more practice with these stimuli in our experiments would have led to a closer replication. Nevertheless, this failure of convergence of the 65 Hz exponents between most of the present 17-stimulus experiments and the 200- and 50-stimulus experiments of West et al. (2000) needs to be investigated further, and indicates that the minimal implementation of CS might not be good enough for scientific purposes in some cases. Moreover it emphasizes the problem that arose in comparing exponents across different protocols: given the regression, practice, and fatigue effects that are ubiquitous in psychological experiments, and the extraordinary cross experiment and cross-individual precision of CS within a given protocol, perhaps not only response scales but even protocols must be standardized in order to achieve the desired level of reproducibility of experiments in psychophysics.

The numbers of valid subjects listed in **Table 2** are relevant to the question of standardization of the protocol. There is a clear trade-off of precision against efficiency in CS, as in any other experimental method. For the 52-stimulus protocol only four of a total of 54 (7.4%) 500 and 5000-Hz runs had *r_RP_*^2^ < 0.67, and the recalibration runs for those frequencies had no such runs whatsoever. On the other hand, for the 17-stimulus protocol, 39 of the total of 205 (19.0%) 65, 500, and 5000-Hz runs, and 12.7% of the corresponding recalibration runs, failed to meet our fairly liberal criterion. For scientific purposes even the 52-stimulus protocol is relatively quick, gives adequate precision, and probably represents a good compromise between efficiency and precision. The numbers just listed, however, make it clear that, although ideal for the clinic, the 17-stimulus protocol will result in a fair number of imprecise measurements, and that measures should be taken to improve results when this occurs. Thus, in our opinion the best approach for the clinic would be to build the psychophysical function in stages and measure *r_RP_*^2^ for each stage (possibly with decreasing numbers of trials) until the desired precision is obtained, similarly to the way the psychometric function is built up using adaptive techniques (e.g., Macmillan, 2002). This approach could also be used for scientific purposes, with usability of results based on achieving a particular criterion of precision. Further research needs to be done to ascertain whether this approach limits the regression, practice and fatigue effects any further than has been achieved in the present study.

**FIGURE 5:**
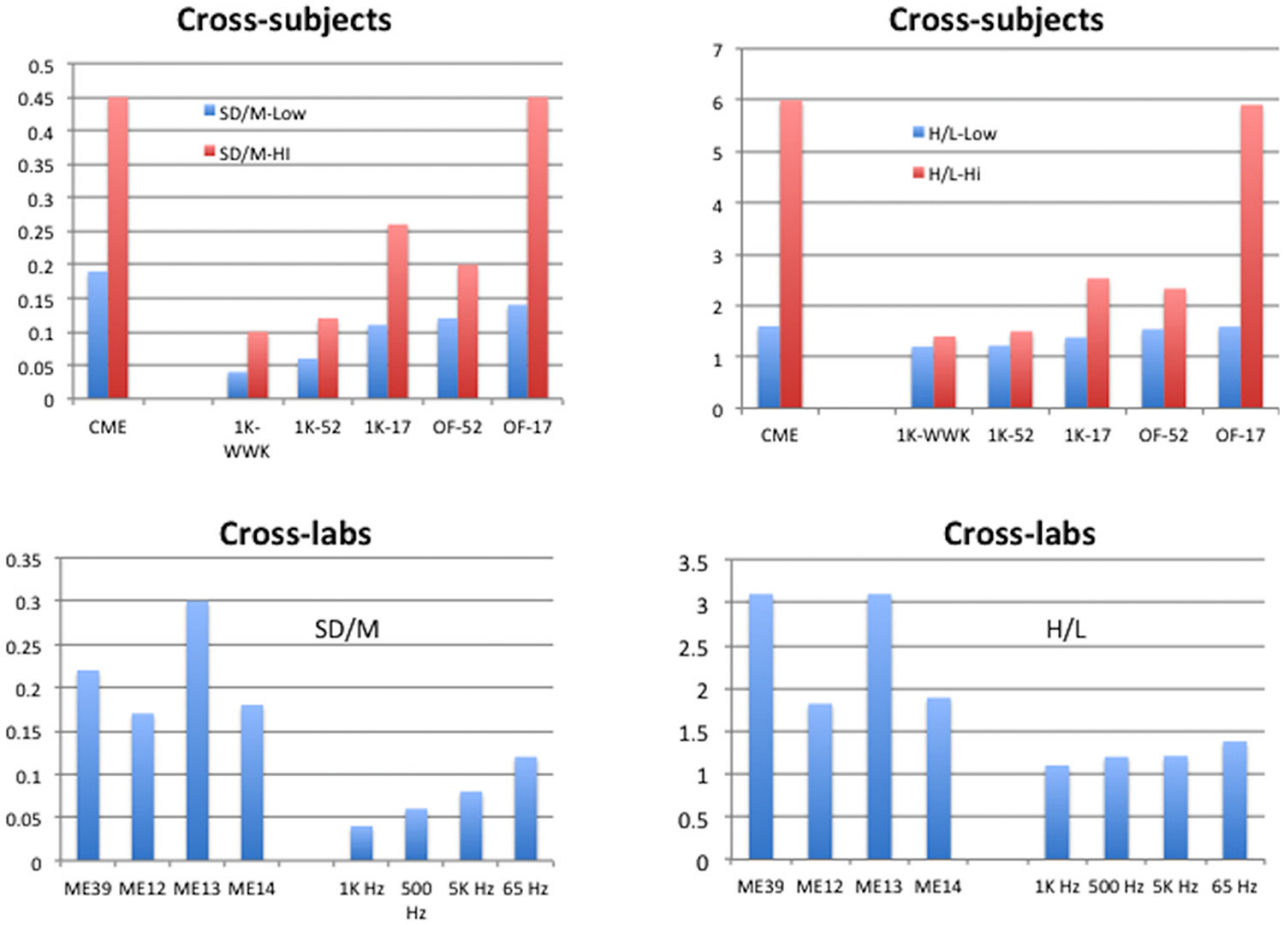
**Summary of results for Cross-subject and Cross-lab indices of performance.** Cross-subjects graphs display the highest and lowest values, across the studies available, of the standard deviation of exponents from the individual participants in each study divided by the mean of those exponents (*SD/M*, **upper left**), or the highest and lowest values, across the studies available, of the ratio of highest to lowest exponents given by the individual participants in each study (*H/L*, **upper right**). The Cross-labs graphs display the *SD/M* of mean exponents **(lower left)** or the *H/L* of mean exponents **(lower right)** from the studies available. Conventional Magnitude Estimation (CME) for 1 kHz stimuli from Table 1 of West et al. (2000); ME12: conventional ME of 1 kHz pure tones for 12 studies from Stevens (1956); ME13: conventional 1 kHz ME for 13 studies from the lab of Ward; ME14: conventional 1 kHz ME for 14 studies from Marks (1974b); ME39: conventional ME across all 39 studies ME12, ME13, ME14; 1K-WWK: 1 kHz constrained scaling (CS) from West et al. (2000); 1K-52: 1 kHz CS from current study with 52 judgments; 1K-17: 1 kHz CS from current study with 17 judgments; OF-52: Other Frequencies from current study with 52 judgments; OF-17: Other Frequencies from current study with 17 judgments; 1 KHz: CS from current study, nine exponents from 1 KHz Recalibration 1 (feedback); 500 Hz: CS from current study, seven exponents from 500 Hz judgments (no feedback); 5 KHz: CS from current study, six exponents from 5 KHz judgments (no feedback); 65 Hz: CS from current study, five exponents from 65 Hz judgments (17-stimulus only, no feedback).

**FIGURE 6:**
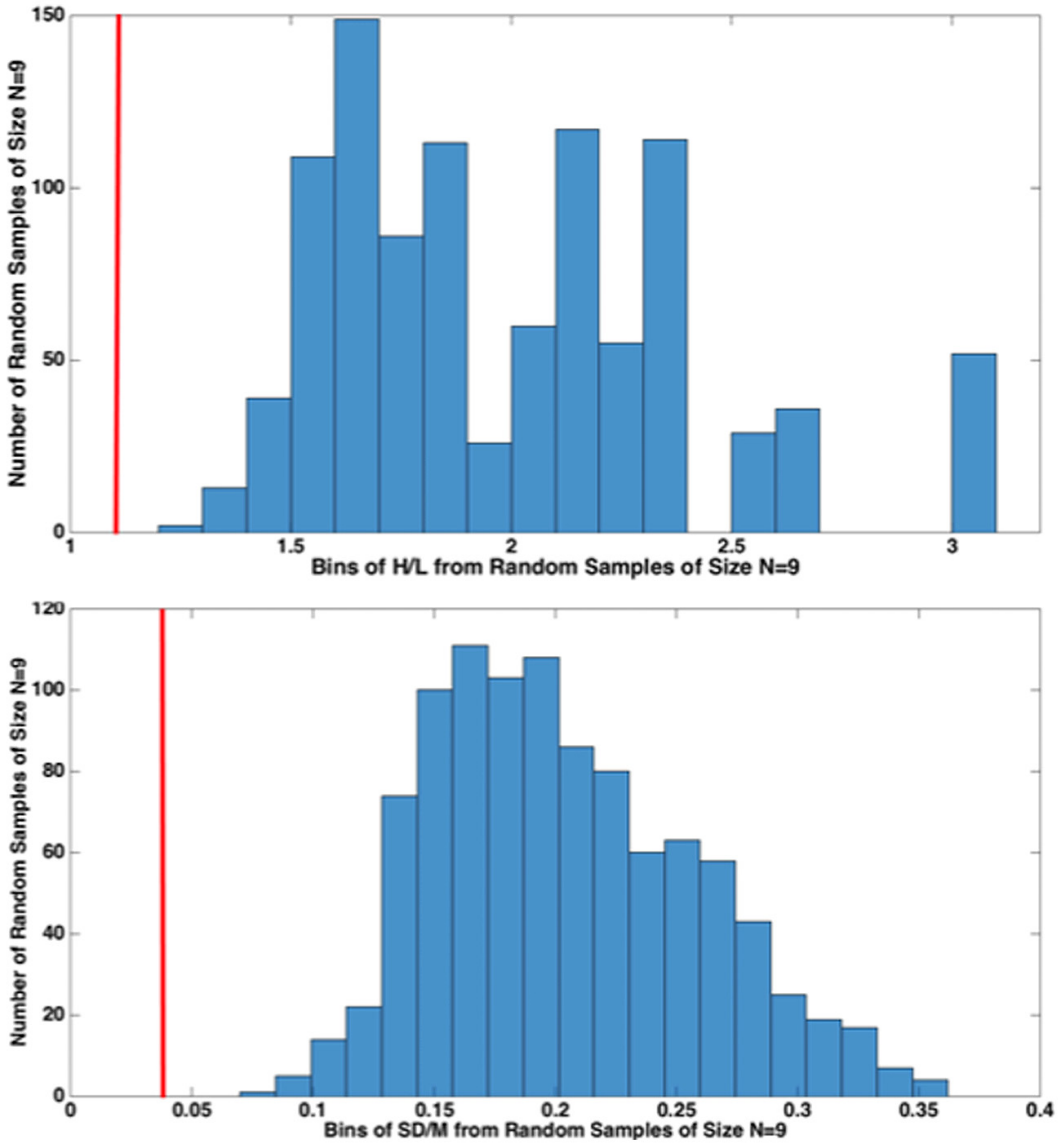
***High/Low* (*H/L*) and *SD/M* comparisons of nine 1000-Hz pure tone exponents from our nine different, Recalibration 1, studies with 1000 random samples of size *N* = 9 from the 39 studies from various labs (histogram), 26 from Marks (1974b) and 13 from Ward’s lab.** The red vertical line (cf. 1 KHz in **Figure 5**) indicates that the value of each statistic for CS falls outside of the distribution of values from the 1000 random samples from the 39 conventional ME studies. This resampling procedure indicates that it is highly unlikely that our sample of nine CS exponents came from a random sample from the population of conventional ME scaling exponents.

**Figures 5** and **6** summarize the *SD/M* and *H/L* statistics across laboratories and protocols for the present studies and compare them to those of conventional techniques as well as to the comparable statistics for individuals reported by West et al. (2000). The aim here is to display the range of *SD*/*M* and *H/L* statistics across different sets of studies in order to make the comparisons meaningful in a broader context. In **Figure 5** the cross-subjects panels display the highest and lowest values of the two statistics found for individual participants in the various studies indicated. The values for conventional magnitude estimation (CME) are taken from Table 1 in West et al. (2000) and represent studies by a wide variety of researchers attempting to use conventional ME. The cross-labs panels in **Figure 5** compare the *SD/M* and *H/L* statistics for the present experiments to several different subsets of the set of 39 conventional ME of loudness of 1000 Hz pure tones studies we mentioned in the Sections “Data Analysis” and “Results.” One subset consists of 12 studies from Stevens lab, one of 13 separate studies from Ward’s lab, and the other of the remaining 14 studies from various labs and summarized by Marks (1974b) and West et al. (2000; **Table 1**). We made these comparisons because the three subsets represent different versions of cross-lab results with the same nominal stimulus set and methods but very different general approaches. The Stevens subset is of course from the lab that popularized the conventional ME technique and represents results that were published in a single paper aimed at validating the technique. The Ward subset represent a group of studies from the same lab but run by different research assistants at different times for different purposes. Finally, the remaining studies taken from Marks (1974b) represent studies from a wide variety of different labs and researchers.

**Figure 6** compares the results from the present nine, Recalibration 1, 1000-Hz pure-tone cross-lab studies (red vertical line) to 1000 random samples (*N* = 9) of exponents from the total of 39 conventional ME studies considered. We did this to reject the idea that our nine exponents are simply a random sample of exponents from the “true” distribution of ME exponents (which we assumed to be roughly that of the 39 conventional ME exponents). **Figure 6** shows that the results of our study lay far outside the distribution of same-size random samples from that distribution, lending weight to the idea that CS indeed improves the cross-lab scaling results, at least as measured by *SD/M* and *H/L*.

Overall, both figures demonstrate that the present CS experiments and those of West et al. (2000), despite substantial differences in lab equipment, observers, and numbers of training and test trials, achieved across-laboratory reproduction of quantitative results substantially superior to those obtainable with conventional techniques. Other modifications of conventional direct scaling techniques also potentially could yield superior reproducibility, e.g., the CR-100 scale of Borg and Borg (2001) or the master scaling technique of Berglund (1991). Borg’s approach, in particular, could be adapted to the CS technique with improved results.

## Conclusion

We believe that one or more of these techniques should be adopted by convention in order to create reproducible canonical scales of sensory and other stimuli. Moreover, our 17-stimulus protocol experiments demonstrate that accuracy comparable with the best of conventional techniques, which often require 100s of judgments per condition, can be obtained in CS for most subjects with only 17 training judgments and 17 calibration plus 17 test judgments per test condition. Moreover, in the clinic the measurement series could be limited to the specific data required for treatment. For example, for a tinnitus sufferer, a training series (at 1-KHz if the sufferer has good hearing there) and a measurement of the tinnitus loudness (silence interleaved with recalibration trials) using the 17-judgment protocol might give adequate data for treatment. This protocol would probably require only about 10 min to administer and would avoid the bulk of fatigue and boredom effects even for those with health challenges. Given the continuing importance of the measurement of sensation, particularly in the clinic (e.g., Borg, 1998; Ward and Baumann, 2009), and the increasing importance of rigorously measuring subjective reports regarding the experiences of consciousness (e.g., Varela and Shear, 1999; Thompson et al., 2005; Fazelpour and Thompson, 2015), the adoption of a standard scaling technique possessing high efficiency and precision is long overdue. We assert that CS, or a variant thereof, is an excellent candidate for that standard.

## Conflict of Interest Statement

The authors declare that the research was conducted in the absence of any commercial or financial relationships that could be construed as a potential conflict of interest.
